# Faecal Microbiota Transplantation as an Adjuvant Treatment for Extraintestinal Disorders: Translating Insights from Human Medicine to Veterinary Practice

**DOI:** 10.3390/vetsci12060541

**Published:** 2025-06-03

**Authors:** Alice Nishigaki, Julian R. Marchesi, Renato L. Previdelli

**Affiliations:** 1 Department of Comparative Biomedical Sciences, Royal Veterinary College, 4 Royal College Street, London NW1 0TU, UK; anishigaki20@rvc.ac.uk; 2 Division of Digestive Diseases, Department of Metabolism, Digestion and Reproduction, Imperial College London, St. Mary’s Hospital, London W2 1NY, UK; j.marchesi@imperial.ac.uk

**Keywords:** faecal microbiota transplant, gut microbiome, veterinary medicine, microbiome therapy, extraintestinal disease, gastrointestinal disease

## Abstract

Faecal microbiota transplantation (FMT) has emerged as a transformative therapy in human medicine, particularly for managing recurrent *Clostridioides difficile* infections and other gastrointestinal (GI) disorders. Beyond the GI tract, FMT has shown potential in addressing extraintestinal conditions in people, including metabolic, immune-mediated, dermatological, neurological, and infectious diseases. Research in people has highlighted its efficacy in decolonising multidrug-resistant organisms in infection, mitigating autoimmune diseases, and improving outcomes in metabolic disorders such as obesity and diabetes. Furthermore, FMT has also been linked to enhanced responses to immunotherapy in cancer and improved management of hepatic and renal conditions. These findings underscore the intricate connections between the gut microbiome and systemic health, opening novel therapeutic avenues. In veterinary medicine, while FMT has demonstrated benefits for GI disorders, its application in extraintestinal diseases remains largely unexplored. Emerging evidence suggests that conditions such as atopic dermatitis, chronic kidney disease, immune-mediated diseases, and behavioural disorders in companion animals could benefit from microbiome-targeted therapies. However, significant gaps in knowledge persist, particularly regarding the long-term safety and efficacy for veterinary applications. This review synthesises findings from human medicine to assess their relevance for veterinary applications and future research.

## Simple Summary

Faecal microbiota transplantation (FMT) is a medical procedure in which stool from a healthy donor is transplanted into the gastrointestinal tract of a recipient to restore a balanced population of gut microbes. FMT has become a transformative treatment, especially for recurrent gut infections in humans, but its benefits extend beyond the digestive system too. FMT has also shown potential in treating a variety of conditions outside of the gastrointestinal tract, including metabolic disorders (such as obesity and diabetes), immune-mediated diseases, skin conditions, neurological disorders, and improving responses to cancer treatments. Studies have highlighted how restoring a healthy balance of gut bacteria can positively influence overall health, supporting the idea that the gut microbiome plays a crucial role in many bodily functions. In veterinary medicine, FMT has been used to treat gastrointestinal issues in small animals, but its potential for addressing other diseases remains largely unexplored. Emerging evidence from research in people and animal models suggests that conditions like allergic skin disease, liver and kidney disease, immune disorders, and behavioural issues in pets could benefit from microbiome-based therapies. However, there are still many unknowns regarding the safety and long-term effects of FMT in animals. This review presents the latest applications of FMT in veterinary medicine and discusses its applications within human medicine considering possible insights for its use to treat extraintestinal disorders in companion animals. With further research, FMT could expand treatment options for both gastrointestinal and non-gut-related diseases in pets.

## 1. Introduction

Faecal microbiota transplantation (FMT) is an innovative treatment which has been increasingly studied in people and domestic dogs. Despite interest in the practice over recent years, the first records of faecal transplantation date back to 4th century China, where “yellow soup” was applied in cases of severe food poisoning and diarrhoea in people [1], and has been frequently used in livestock systems to treat GI diseases [2,3]. FMT aims to treat disturbed gut microbiota and “correct” dysbiosis by transplanting material from healthy donor stool into a recipient’s gastrointestinal tract (GIT) [4]. It is a recommended treatment option in people with recurrent or refractory *Clostridioides difficile* infection, particularly when standard antibiotic therapies have failed to achieve sustained resolution [5]. FMT has demonstrated high efficacy in restoring gut microbial diversity, reducing recurrence rates, and improving clinical outcomes in patients with multiple episodes of *C. difficile* infection [6]. As a result, it has been incorporated into clinical guidelines by major health authorities, including the National Institute for Health and Care Excellence (NICE) as a viable therapeutic option for managing difficult-to-treat *C. difficile* infections [7]. In recent years, FMT has also been increasingly used in domestic canine species. Until recently, the lack of treatment regulation and clinical guidelines has meant that the application of FMT in widespread clinical veterinary practice has been hindered. However, the recent publication of clinical guidelines for the use of FMT in companion animals represents a significant advancement in veterinary medicine and acknowledges its potential therapeutic effects in domestic animals. These guidelines provide detailed recommendations on the preparation and administration of FMT, as well as criteria for donor selection, ensuring a standardised approach to this innovative therapy [8]. By offering a clear framework, veterinary clinicians are empowered to safely and effectively integrate FMT into the management of gastrointestinal diseases in companion animals and expanding therapeutic options. Since 2016, the number of publications reporting FMT in dogs has increased considerably from previous years, and an increasing number of clinical trials have been undertaken to investigate the applications of FMT in dogs, mostly within the context chronic and acute gastrointestinal (GI) diseases [9,10,11,12,13,14]. Nevertheless, the use of FMT to treat extraintestinal disorders in veterinary medicine has not been as extensively explored as in human medicine, presenting an opportunity for further investigation and application in companion animals. The potential for FMT to treat extraintestinal diseases in companion animals is largely theoretical, necessitating rigorous scientific evaluation to determine its safety, efficacy, and optimal methodologies.

A key challenge in veterinary FMT is the adaptation of human-based methods for use in companion animals. Ensuring long-term safety is a critical concern. In human medicine, adverse effects of FMT are rare but have included transient GI symptoms and, in isolated cases, severe bacteraemia, perforations and death [15]. Veterinary studies must assess whether similar risks exist in dogs and cats. Further research is also needed to determine the optimal administration routes (e.g., oral capsules vs. enemas) and long-term effects of microbiome manipulation in veterinary species. While the general principle of FMT is to restore gut microbial balance, the exact mechanisms remain incompletely understood, particularly in veterinary species. In people, FMT has been shown to influence systemic inflammation, metabolic pathways, and immune regulation [16]. However, whether these same mechanisms apply to companion animals remains speculative and requires further research. The historical lack of regulatory oversight in veterinary medicine raises additional concerns, such as screening protocols for donor stool and appropriate dosing strategies. The introduction of new clinical guidelines has significantly addressed some of these concerns by establishing more standardised screening protocols for donor stool, ensuring safer and more consistent dosing strategies for dogs and cats [8]. These guidelines help to mitigate risk by providing evidence-based frameworks for veterinarians to follow, promoting more controlled and safer use of FMT.

The objective of this review is to examine how insights from human medicine can inform future veterinary research on the applications of FMT for extra-intestinal conditions in dogs and cats. By leveraging the more advanced understanding and clinical applications developed in human healthcare, veterinary medicine stands to benefit from an accelerated translational approach. Cross-species comparisons such as these offer mutual benefits for the advancement of novel therapeutics and disease models, while also guiding future investigations into the mechanisms and efficacy of microbiome-based interventions in companion animals. Ultimately, integrating findings from human research can help to guide future veterinary research, contributing to a more holistic and collaborative model of biomedical science.

## 2. Methods

To identify the relevant literature on gut microbiome alterations in companion animals across various disease states, a structured search was conducted using electronic databases, including PubMed and CAB Abstracts. Keywords and Boolean operators were tailored to capture a broad range of studies, including terms such as “*gut microbiome*”, “faecal microbi* transplant*”, “*extraintestinal disease*”, “*companion animals*”, “*dog*”, “*cat*”, and “*human*”. Searches were limited to English-language publications and peer-reviewed articles. Articles were screened manually in two stages: initially by title and abstract, followed by full-text review to identify studies relevant to the use of FMT for the treatment of extraintestinal diseases in humans, dogs, cats, or mouse models. Additionally, reference lists of selected papers were subject to manual review to identify further relevant literature.

## 3. Discussion

### 3.1. Current Applications of FMT in Domestic Dogs

FMT has emerged as a potential therapeutic option for chronic and acute GI diseases in canine patients, with limited research in cats. Promising results have been reported, such as the rapid improvement of Canine Inflammatory Bowel Disease Activity Index scores in dogs with chronic large bowel diarrhoea, and the reduction in clinical signs in cases of acute and refractory chronic enteropathy [9,11,12]. However, not all findings are consistent, with some studies reporting no significant differences between FMT and alternative treatments, such as sham procedures, probiotic therapy, or psyllium husk administration in conditions like acute haemorrhagic diarrhoea syndrome and inflammatory bowel disease (IBD) [12,13,14,17]. These inconsistencies may stem from the historical lack of standardised FMT protocols, leading to variations in preparation methods, dosage, and administration techniques. While many studies use a saline-based slurry with fixed ratios [10], others rely on more arbitrary formulations, basing dosage on body weight, breed size, or texture of faecal slurry [9,11]. Some protocols also incorporate colon cleansing prior to FMT [13]. The recent development of clinical guidelines for companion animals is a critical step forward, providing a framework for standardised FMT practices that will likely improve clinical outcomes and facilitate broader adoption of this therapeutic approach in veterinary medicine [8].

FMT has emerged as a transformative therapy in human medicine, particularly for managing *C. difficile* infection and various GI disorders such as Crohn’s disease and ulcerative colitis. Its success has spurred interest in exploring its potential beyond the GI tract, with promising research finding a role of the gut microbiome in metabolic, immune-mediated, and neurological diseases (Table 1) [18,19,20,21]. These findings highlight the diverse systemic connections of the gut microbiome, emphasising its potential as a versatile therapeutic target across multiple organ systems. Studies concerning the application of FMT in companion animals are currently lacking, though preliminary research, primarily in rodent models, has been conducted (Table 2). The publications included in Table 1 and Table 2 were included based on their relevance to the use of FMT in treating extraintestinal disorders, both in human and animal research contexts. The selection criteria focused on studies that explored the efficacy, safety, and methodologies of FMT in disorders outside of the GIT. It becomes evident that there is a significant gap in veterinary medicine regarding the application of FMT for extraintestinal disorders. Table 1, which includes human research studies, presents a broad range of applications, with numerous clinical and experimental studies addressing the use of FMT in treating autoimmune conditions, metabolic diseases, and systemic infections, among other extraintestinal disorders. This wealth of human research highlights the growing recognition of FMT’s potential in managing diseases beyond the gastrointestinal system. In contrast, Table 2 shows a noticeable lack of similar research in companion animals. The scarcity of studies in companion animals highlights the limited research conducted within veterinary medicine on the potential of FMT for treating extraintestinal disorders. The inclusion of rodent models in Table 2 serves to indicate the potential for future research directions in companion animals, suggesting that although veterinary studies are limited, animal research overall has laid a foundation for exploring the broader applications of FMT in veterinary practice. While these models have contributed significantly to foundational knowledge, it is important to acknowledge that murine physiology is more closely aligned with humans and may not reliably represent responses in species such as dogs. More studies are urgently needed in companion animals to bridge the gap between the extensive human literature and the relatively underexplored veterinary context.

**Figure 1:**
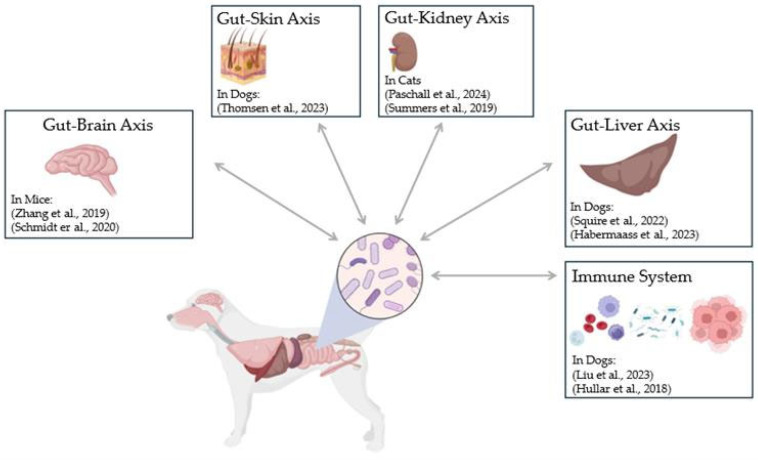
Potential relationships between the gut microbiome and extra-intestinal body systems as described in different veterinary species [37,38,41,42,43,44,45,46,47]. Figure created in Biorender® (ver. 04).

As research continues to elucidate the intricate connections between the gut microbiome and systemic health, FMT holds promise for addressing extraintestinal conditions in companion animals, where studies have investigated various gut-organ axes [37,38,41,42,43,44,45,46,47] (Figure 1). Opportunities exist in veterinary species where conditions such as allergic skin disease, obesity, and even behavioural disorders, such as anxiety, could be targeted through microbiome restoration. Despite this potential, substantial gaps in knowledge persist, particularly regarding the long-term safety of FMT, and its efficacy for non-GI conditions.

The recent development of clinical guidelines for FMT in companion animals represents a critical step forward, providing a framework for standardised practices that will likely improve clinical outcomes [8]. However, these guidelines remain focused on GI diseases, and there is no veterinary consensus on whether FMT could be beneficial for extra-intestinal disorders, as seen in human research. While anecdotal reports and preliminary data suggest that FMT could play a role in companion animal conditions such as allergic skin disease, obesity, and behavioural disorders, definitive studies are lacking. Future veterinary research should investigate whether microbiome-targeted therapies could address extraintestinal diseases in animals, as observed in human medicine.

### 3.2. Dermatological Disorders

In veterinary medicine, current treatment options for conditions such as canine atopic dermatitis (CAD) include corticosteroids, antihistamines, immunosuppressants, and biologic drugs [48]. These treatments provide temporary relief but often fail to address the underlying causes of the condition. Long-term use of some therapies can also result in adverse effects, including immunosuppression. Additionally, many animals exhibit variable responses to these treatments, making management challenging and often requiring a multimodal approach. The link between the gut microbiome and the skin in dogs has been demonstrated, with one study finding that dogs with CAD exhibited clear gut microbiota dysbiosis alongside skin dysbiosis [41]. In healthy dogs, the dominant gut bacterial phyla included Firmicutes, Bacteroidetes, Proteobacteria, and Fusobacteria. However, CAD-affected dogs showed a significant reduction in Fusobacteria, with a concurrent increase in potentially pathogenic bacteria such as *Escherichia*/*Shigella* spp. and *Clostridium sensu stricto*. Beta diversity of the gut microbiome significantly differed between healthy animals and those with mild CAD, and three phyla (Bacteroidetes, Actinobacteria, and Firmicutes) were positively correlated with Canine Atopic Dermatitis Extent and Severity Index total score, while Acidobacteria, Proteobacteria, and Synergistetes were inversely correlated. These findings further support the concept of a gut–skin axis in dogs with CAD. Fusobacterium, a common commensal in carnivores and non-carnivores, was also significantly reduced in CAD dogs [41]. In people, Fusobacteria have been associated with GI diseases [49]; however, its role in dogs appears to differ. In canine gut microbiota, its presence is correlated with increased short-chain fatty acids (SCFAs) such as butyrate and propionate [50,51]. Higher levels of these SCFAs in humans has been shown to reduce the risk of atopy in early life; therefore, it can be proposed that the reduced abundance of these SCFA-producing bacteria in CAD dogs may contribute to disease pathology. The relationship between CAD and IBD in dogs mirrors the connection between atopic dermatitis and IBD in humans, suggesting the utility of Fusobacteria as a potential biomarker for both conditions.

FMT has shown benefits in dermatological disease in humans, such as effective treatment of moderate-to-severe atopic dermatitis, with significant reductions in symptom severity observed after multiple transplants [23]. In another study, patients experienced an improvement in clinical atopic dermatitis scores and decreased reliance on topical corticosteroids following FMT [24]. A recent study in dogs also demonstrated that a single oral FMT can significantly decrease Canine Atopic Dermatitis Extent and Severity Index and pruritus scores, indicating its potential efficacy in managing the disease [36]. Modulating the gut microbiome through FMT offers a potential adjunctive strategy for managing CAD, by targeting systemic inflammation and immune dysregulation, addressing some of the limitations of traditional treatment options. The proposed mechanisms of action include restoring gut microbial diversity, thereby enhancing the production of SCFAs, which play a pivotal role in maintaining gut barrier integrity, regulating immune responses and modulating systemic inflammation [52,53]. Smith and colleagues (2013) discuss the role of microbial metabolites, particularly SCFAs, in regulating colonic T-regulatory cell homeostasis. This study suggests that SCFAs may directly impact T-regulatory cell differentiation by acting on the GPR43 receptor, a free fatty acid receptor expressed on immune cells. This interaction promotes the development of T-regulatory cells that help to maintain immune homeostasis and prevent autoimmune conditions [52].

The study by Sugita and colleagues (2023) on CAD represents the first exploration of FMT for an extraintestinal disease in veterinary species, demonstrating that by restoring microbial balance, FMT may improve treatment outcomes in CAD patients, while reducing reliance on drugs with significant side effect profiles [36]. Ongoing clinical research continues to explore the efficacy of FMT in treating CAD, aiming to provide more comprehensive and sustainable management options for this chronic condition. The successful application of FMT as a novel approach to aid the treatment of dermatological conditions in veterinary medicine could pave the way for further investigation into its broader impact on extraintestinal diseases in companion animals, offering new opportunities for treatment approaches in conditions traditionally challenging to manage.

### 3.3. Infection, and Susceptibility to Multidrug-Resistant Organisms

In people, FMT has demonstrated significant potential in addressing a patient’s susceptibility to multidrug-resistant organisms (MDROs) [54]. By restoring the disturbed microbiome with a diverse microbial community from healthy stool donors, FMT has been proposed as a method to reduce intestinal colonisation by MDROs, thereby preventing infections and reducing their clinical impact within and outside of the GIT [55,56,57,58]. Studies have shown a decrease in colonisation rates of MDROs in patients with recurrent *C. difficile* infection [59], faster MDRO decolonisation, and protection against recurrent infections following FMT [60]. Notably, extended-spectrum beta-lactamase (ESBL)-producing strains were replaced by non-ESBL strains following FMT [60], and carbapenemase-producing Enterobacteriaceae were successfully decolonised [61]. Moreover, FMT has been associated with the downregulation of resistance genes, such as VanA in people [62].

FMT has also shown promise in managing persistent urinary tract infections (UTIs) in people, particularly those caused by MDROs. It has been shown to significantly decrease the frequency of recurrent UTIs and improve the antibiotic susceptibility profile of UTI-causing organisms [25,63]. These findings highlight FMT’s potential as a cost-effective alternative to managing antibiotic-resistant infections. Additionally, there have been reports of FMT being used to successfully treat sepsis and multiorgan dysfunction syndrome resulting from sepsis in people [26,27]. In veterinary medicine, similar strategies could be applied to combat antimicrobial resistance, a growing global concern. In dogs and cats, FMT could provide an innovative approach to managing conditions like antibiotic-refractory diarrhoea and should also be considered for use in the context of extra-intestinal recurrent infections such as UTIs that contribute to lower urinary tract disorders, which are a common problem in domestic felines [64]. Special consideration should be given to the use of FMT in immunocompromised animals, where MDROs pose a significant risk. FMT within these clinical scenarios presents the possibility to help downregulate resistance genes, which could also help to slow the spread of resistance within domestic animals, ultimately aligning with WHO One Health objectives [65].

### 3.4. Hepatic Disorders

FMT has been shown to improve symptoms of hepatic encephalopathy in people [28,66], with sustained clinical responses reported up to 20 weeks post-transplant in another study [29]. In non-alcoholic fatty liver disease (NAFLD), FMT improved small intestinal permeability [67], necro-inflammatory histology, and hepatic gene expression related to inflammation and lipid metabolism [68]. Additionally, patients with cirrhosis who were treated with FMT experienced improved Child–Turcotte–Pugh scores, reduced pro-inflammatory cytokines [69], and lowered plasma ammonia levels post-FMT [70]. Although not directly applicable to the domestic animal population, FMT has also demonstrated efficacy in human trials for hepatitis B, and has shown superior outcomes compared to tenofovir alone in managing liver failure caused by the virus, highlighting its potential for addressing viral hepatic diseases [71,72]

In companion animals, hepatic disease poses significant challenges. Emerging evidence from human studies accentuates the potential of targeting the gut-liver axis to manage liver conditions. The gut–liver axis is mediated by the portal vein, bile acids, and systemic circulation, linking disturbances in the gut microbiome to liver injury [73]. Such disturbances, often caused by antibiotics, diet, or disease, can lead to increased intestinal permeability and the translocation of microbial products, such as lipopolysaccharide, to the liver, triggering inflammation, oxidative stress, and immune responses. Common liver diseases in dogs and cats include chronic hepatitis, hepatic lipidosis (in cats), portosystemic shunts (PSS) with systemic toxin accumulation and hepatic encephalopathy, and drug- or toxin-induced liver injury [74]. Studies have shown that gut dysbiosis is prevalent in dogs undergoing medical treatment for PSS [44], while altered gut microbial composition is also observed in dogs with cholestasis [45]. Dogs with chronic hepatobiliary disease have been shown to have similar changes in the composition of the gut microbiome to those changes seen in dogs with acute diarrhoea (reduction in *Ruminococcaceae* and *Turicibacter*) and chronic enteropathy (reduced *Fusobacteriaceae* and *Bacteroidaceae*, increased *Escherichia*/*shigella*). *Clostridium hiranonis*, an important species involved in the deconjugation of primary bile acids to secondary bile acids in dogs, was also found to be reduced in dogs with chronic hepatobiliary disease [45]. Given that elevated levels of secondary bile acids have been associated with immune regulation in vitro [75], this reduction may suggest a potential role for *C. hiranonis* in maintaining immune homeostasis and gut–liver axis integrity. The authors of this study propose that altered bile quality and flow in cholestatic dogs may contribute to the loss of *C. hiranonis*, potentially exacerbating disturbances in bile acid metabolism. Similar considerations arise in feline hepatic disease. For example, hepatic lipidosis in cats is a condition primarily associated with a negative energy balance, usually caused by anorexia or prolonged catabolism [76]. Studies in people have explored the role of microbiome-mediated development and progression of NAFLD, a condition histologically comparable to hepatic lipidosis in cats [77,78], though no such studies exist in feline populations. In a murine model of NAFLD, gut microbiota removal showed a lowered abundance of bacteria linked to aberrant metabolism and inflammation [79]. In addition, mice with diet-induced obesity given gut microbiota from normal mice showed increased abundances of *Lactobacillus* and *Christensenellaceae* alongside a reduction in steatohepatitis [80]. Investigating gut microbiome alterations in feline hepatic disease may therefore uncover novel diagnostic markers or therapeutic targets and help to clarify the role of host-microbiota interactions in liver health across species.

Parallels with human conditions suggest that FMT could offer novel therapeutic approaches for these issues. For example, in dogs with PSS or severe liver dysfunction, FMT could reduce gut-derived ammonia production, alleviating neurological symptoms associated with hepatic encephalopathy. The anti-inflammatory and immune-modulating effects of FMT may complement traditional treatments for chronic hepatitis, slowing disease progression and fibrosis. Additionally, FMT could support recovery from drug or toxin-induced liver injury by promoting detoxification through microbiome-mediated bile acid metabolism and bile acid signalling pathways [63]. In cases of hepatic lipidosis in cats, which can be histologically compared to NAFLD in people [77,78], FMT could help to alter lipid metabolism and inflammation through enhancing the gut microbiota.

The protective role of SCFAs against toxin-induced liver injury in rats has been explored [81]. Sodium butyrate, in particular, has been shown to safeguard the liver from toxin-induced damage, underscoring the critical role of microbiome-derived metabolites in modulating liver inflammation and injury [81]. FMT offers the potential to restore a balanced gut microbiota, thereby enhancing the production of beneficial SCFAs like butyrate. As such, FMT could represent a promising therapeutic strategy, improving gut–liver interactions and supporting hepatic function, offering a novel approach to managing toxin-induced acute liver failure in companion animals.

### 3.5. Neurological Disorders

Animal models and human studies suggest that modulating intestinal microbiota via FMT can influence psychiatric symptoms, demonstrating both symptom relief when microbiota from healthy donors are transferred to ill recipients and symptom transmission when microbiota from ill donors are transplanted to healthy recipients [82]. This efficacy has been observed across disorders such as depression, anxiety, anorexia, and alcoholism. However, the benefits in clinical studies were often transient, lasting 3–6 months in most cases [82], possibly due to the re-establishment of the recipient’s original microbiota over time, insufficient long-term colonisation of beneficial microbes, or the need for repeated transplants to maintain microbial balance and sustained improvement in clinical signs. The application of FMT for psychiatric disorders in humans is still in its early stages and requires further research to fully understand the mechanisms, long-term efficacy, and safety. However, the promising results observed in recent clinical trials highlight its potential as a novel therapeutic option [83,84,85]. It is important to note, however, that these studies predominantly involved patients with IBD or irritable bowel syndrome, conditions that are both physically and mentally taxing [86,87]. Therefore, the observed improvements in psychiatric symptoms may not solely be due to FMT, but, rather, a result of the alleviation of gastrointestinal symptoms, leading to better mental health outcomes. However, studies in mice models have shown promising results [37,38]. Elevated NLRP3 mRNA levels have been demonstrated in the blood of people with depression [88]. Based on this premise, faecal microbiota from NLRP3 knockout mice was used for FMT to model a non-depressed state. In mice subjected to chronic unpredictable stress, FMT from NLRP3 knockout donors significantly ameliorated astrocyte dysfunction [37], demonstrating its potential to mitigate stress-induced neurological dysfunction. In another study, FMT was shown to prevent anxiety like behaviours in rats with spinal cord injuries [38]. These findings highlight the therapeutic potential of FMT for modulating gut–brain interactions and suggest a promising avenue for managing anxiety and depression-related conditions in dogs. In particular, FMT could be explored as a complementary treatment for dogs experiencing chronic stress or painful conditions, such as acute spinal cord injuries, alongside existing therapeutic strategies [89].

In humans, the benefits of FMT have also been demonstrated in the treatment of neurological disorders, including epilepsy. Epileptic patients receiving FMT experienced an incidental reduction in seizures, with some showing no recurrence over extended follow-ups [30,90]. These findings suggest a role for the gut–brain axis in seizure modulation. In veterinary medicine, this research could have important implications for treating epilepsy in companion animals. Given the growing understanding of the gut–brain axis, FMT may offer a novel therapeutic avenue for managing seizures and other neurological disorders in animals, potentially improving outcomes where traditional treatments are less effective.

### 3.6. Metabolic Disorders

FMT has shown promise for managing metabolic disorders in people. Studies have demonstrated improvements in glycaemic control in cases of obesity and diabetes mellitus [91], reduced insulin requirements [31], and halted disease progression in recently diagnosed type-1 diabetics by preserving endogenous insulin production [32]. FMT has also been linked to weight loss in obese individuals [92]. Analysis revealed a statistically significant reduction in body mass index following FMT, with a more notable decrease in patients with a body mass index over 30. This reduction was sustained at 3–6 months and 9–12 months, remaining statistically significant for patients with a body mass index greater than 25 [92]. The transmissibility of obesity has also been demonstrated in mouse models, where FMT from obese donors resulted in weight gain in the recipient mice [39]. Interestingly, the composition of the gut microbiome has also been shown to influence feeding behaviour and dietary preferences in animal models [93,94]. In people, lower bacterial richness in the gut microbiome has also been associated with higher circulating leptin concentrations [95], and modifications in gut microbiota have been found to impact ghrelin signalling pathways [96]. These findings emphasise the potential role of the gut–brain axis in regulating feelings of hunger and satiety. Furthermore, it has been shown that *Actinobacteria* abundance is substantially higher in faeces collected from obese versus lean dogs [97]. Another study also revealed that lean dogs had a gut microbiome dominated by *Firmicutes*, whereas obese dogs’ microbiome consistent predominantly of *Proteobacteria* [98]. The association of gut microbiome and weight loss has also been explored in a study by Kieler et al., (2017) who reported that dogs showing a faster weight loss rate presented a low faecal abundance of *Ruminococcaceae* [99]. Similarly, a study in cats also demonstrated that lean cats display higher proportions of *Firmicutes* and lower *Bacteroidetes* compared to obese cats [100]. Given the growing prevalence of obesity in canine and feline populations, future treatments may involve strategies to modulate gut microbiota through interventions like FMT, potentially influencing feeding behaviour and supporting weight management.

The role of the gut microbiome in the development of diabetes mellitus is also demonstrated in both animal models and human studies. Notably, a mouse study reported that transferring the faecal microbiome from non-obese diabetic mice, which develop a type-1 diabetes mellitus (T1DM)-like disease, to non-obese resistant mice induces T1DM, highlighting the diabetogenic potential of the gut microbiome [40]. In people, alterations in gut microbiota composition in diabetes mellitus have been noted. Patients with T1DM exhibit increased levels of Proteobacteria, Actinobacteria, and Bacteroidetes, alongside a reduction in butyrate-producing bacteria, which are essential for maintaining gut barrier integrity [101]. T1DM has also been associated with elevated inflammatory markers and impaired differentiation of regulatory T-cells, reducing immune tolerance to pancreatic β-cells and promoting autoimmunity [101]. Similar reductions in butyrate-producing bacteria are observed in patients with type-2 diabetes mellitus (T2DM) [102]. SCFAs, especially butyrate and propionate, are crucial in intestinal glucose metabolism and maintaining gut health. In rats, research has shown that sodium butyrate enhances AMP-activated protein kinase phosphorylation, boosts Glucagon-like peptide-1 secretion, and promotes the production of colonic mucin and tight junction proteins [103]. These effects collectively strengthen the intestinal barrier and help to alleviate insulin resistance [103]. Given that chronic inflammation and impaired gut barrier function have been implicated in the development of T2DM and insulin resistance in both humans and animal models [104,105,106,107,108], targeting SCFA production through FMT offers a promising therapeutic strategy for managing metabolic disorders such as DM. In dogs with diabetes mellitus, an overexpression of *Clostridiaceae* and a marked increase in *C. difficile* in the gut microbiome have been reported [109], which may contribute to common complications such as chronic diarrhoea in the diabetic dog [110]. Research in rats has also shown that untreated diabetes mellitus promotes intestinal microbial overgrowth, exacerbating dysbiosis and potentially driving specific microbial shifts, such as the overexpression of *C. difficile*. FMT has proven effective in addressing *C. difficile* in people [111], highlighting its potential as a therapeutic option for other species, including dogs. In addition, a pilot study has observed microbial changes in the rectal microbiome of diabetic dogs undergoing 12 weeks of insulin therapy, noting an increase in *Bacteroides* linked to improved insulin sensitivity, and a decrease in *Romboutsia* associated with better glycaemic control [108]. Given the growing evidence of microbiome alterations in diabetes mellitus and their impact on disease progression, FMT should be considered as a therapeutic strategy in managing canine diabetes mellitus.

The growing understanding of the gut microbiome’s role in regulating metabolism presents exciting opportunities for non-invasive treatments in veterinary care for conditions such as diabetes mellitus, potentially improving the quality of life and long-term health of companion animals. These approaches could also enhance client compliance by reducing the burden of daily insulin injections. Studies have highlighted that the treatment commitments associated with insulin administration, such as the risk of hypoglycaemic crises, the cost of therapy, and the disruption to daily routines, significantly affect the lives of pet owners with diabetic animals, and can even contribute to the decision to euthanise pets [112]. Recent advances in managing feline diabetes mellitus have led to the development of less invasive options, such as SGLT2 inhibitors, which have been shown to result in excellent owner compliance [113]. This shift toward less invasive management is encouraging, paving the way for exploring FMT as a potential therapeutic option, particularly in easily administered forms like lyophilised oral tablets [114].

### 3.7. Oncological Applications

Changes to the composition of the gut microbiome have been documented in people and dogs with cancer. Colorectal tumours are rarely diagnosed in dogs, but, despite this, it has been shown that dogs with colorectal tumours have distinct faecal microbiota profiles (increased *Enterobacteriaceae*, *Bacteroides*, *Helicobacter*, *Prophyromonas*, *Peptostreptococcus,* and *Streptococcus*; decreased *Ruminococcaceae*, *Slackia*, *Clostridium* XI, and *Faecalibacterium*) [115]. A pilot study also revealed significant differences in microbial populations present in dogs with multicentric lymphoma versus healthy dogs [116], and another study characterised the gut microbiome in dogs with mammary tumours compared to healthy controls [117]. These findings suggest that gut microbiome profiling may hold diagnostic potential, with distinct microbial signatures serving as non-invasive biomarkers for cancer detection, disease monitoring, and potentially guiding therapeutic strategies in canine oncology.

FMT’s emerging role in human oncology, particularly in enhancing the efficacy of immunotherapy, also has significant implications for veterinary medicine. In people, FMT has been used to overcome resistance to anti-PD1 therapy in patients with immunotherapy-refractory melanoma, ultimately improving treatment response [33,118]. In veterinary medicine, immunotherapy is increasingly used for the treatment of cancers such as canine lymphoma and melanoma [119,120]. Thus, a potential application of FMT in oncological disorders is to enhance the effectiveness of chemo- and radiotherapies by modulating the gut microbiota and indirectly improving immune responses [121,122]. Additionally, FMT might play a supportive role in reducing side effects or complications associated with treatments like chemotherapy or radiation, which can disrupt the gut microbiome and compromise immune function [123]. Research suggests that the gut microbiota plays a significant role in modulating the efficacy and toxicity of anticancer therapies, and by restoring a healthy microbial balance, FMT may help alleviate gastrointestinal side effects and support immune function during cancer treatment [124,125]. By leveraging the gut microbiota’s influence on immune modulation, FMT could become a valuable adjunct therapy in veterinary oncology, improving survival rates and quality of life for companion animals undergoing cancer treatment.

### 3.8. Immune Mediated Disorders

Alterations in the gut microbiome have been observed in dogs with immune-mediated disorders [126]. In cases of canine osteoarthritis, the gut microbiome showed an increased abundance of *Megamonas* [127], a genus also reported to be enriched in human conditions such as NAFLD and ulcerative colitis [128,129]. In cats with chronic asthma, rectal swab analyses revealed increased levels of *Atopobiaceae*, *Porphyromonadaceae*, *Clostridiales Family XI*, and *Pasteurellaceae*, while *Helicobacteraceae*, *Veillonellaceae*, *Erysipelotrichaceae*, and *Acidaminococcaceae* were found to be reduced [130]. An increased presence of *Pasteurellaceae* may contribute to the aetiology of feline asthma, given that certain strains within this family can produce histamine [131], a key mediator linked to the heightened airway reactivity that defines the condition. Additionally, the reduced abundance of *Erysipelotrichaceae* could be associated with pro-inflammatory changes, as this family has been linked to the production of butyrate, an SCFA acid known for its anti-inflammatory properties [132].

Emerging evidence in human medicine highlights the potential of FMT in managing autoimmune disorders. A pilot clinical trial in systemic lupus erythematosus patients demonstrated that FMT, administered alongside standard treatment, led to significant reductions in disease activity, serum anti-dsDNA antibodies, and inflammation-related cytokines, while enriching SCFA-producing bacteria in the gut [34]. In another study, an unexpected resolution of idiopathic thrombocytopenic purpura (ITP) was observed in a patient treated with FMT for ulcerative colitis, indicating its broader immunomodulatory effects [133]. It is reasonable to suggest that in veterinary species, equivalent and closely related conditions such as systemic lupus erythematosus, immune-mediated haemolytic anaemia, immune mediated thrombocytopaenia, and immune-mediated polyarthritis could benefit from microbiome-targeted therapies like FMT. In a small cohort of dogs, a pilot study revealed that immune-mediated haematological disease in dogs is associated with alterations of the faecal microbiota [46]. Given the significant influence of gut microbiota on systemic immune regulation, the potential for microbiota disturbance in immune mediated conditions highlights an opportunity for contextualised patient care. Screening the gut microbiota composition in animals presenting with immune-mediated diseases may offer valuable insights into the extent of dysbiosis. This would allow veterinarians to identify candidates who might benefit from adjunctive FMT to restore microbial balance and enhance therapeutic outcomes. However, more research is needed to establish specific microbiome profiles or biomarkers that can reliably predict disease states and therapeutic responses. Understanding the key microbial signatures associated with immune dysregulation will be critical to refining diagnostic tools and tailoring microbiome-based interventions for optimal clinical impact.

The gut microbiome and immune system are closely interconnected, with recent research even suggesting their role in influencing the risk of canine gastric dilatation-volvulus (GDV) [47]. The development of GDV in dogs is influenced by both genetic and environmental factors, with recent work highlighting the role of the gut microbiome and immune system in altering susceptibility to the condition, where mutations in immune-related genes such as TLR5, DLA88, and DRB1 have been associated with increased GDV risk [47]. These mutations can impair immune surveillance, leading to gut dysbiosis that may exacerbate inflammation and contribute to GDV [47]. In healthy dogs, the gut microbiome is diverse, but GDV-prone dogs [134] show reduced microbial diversity and an overabundance of certain phyla, particularly Firmicutes and Actinobacteria, including *Collinsella* [47]. This increase in pro-inflammatory bacteria may disrupt gut barrier function, promoting systemic inflammation and further increasing GDV risk. The immune system’s interaction with these microbial communities is crucial in regulating gut health and preventing disease, suggesting that by targeting microbiome imbalances, through FMT interventions, it could offer potential therapeutic strategies for preventing GDV in genetically predisposed dogs. Understanding the functional roles of specific microbes in GDV pathogenesis is key to developing more effective prevention and treatment options.

### 3.9. Kidney Disorders

In a clinical trial, FMT administration in people diagnosed with chronic kidney disease (CKD) secondary to diabetes and hypertension was shown to reduce disease progression [35]. Several case studies have also reported the use of FMT in patients with CKD; in one patient with membranous nephropathy, FMT was able to ameliorate symptoms and improve renal function [135]. In a study involving two patients with IgA nephropathy, intensive fresh FMT over 6–7 months led to partial clinical remission, with significant reductions in urinary protein levels, increased serum albumin, and stable kidney function. These improvements coincided with enhanced gut microbiota diversity, including a decrease in Proteobacteria and an increase in beneficial *Prevotella* [136].

CKD is a prevalent and progressive condition in aging cats and dogs, characterised by the gradual loss of kidney function. In cats, CKD is one of the most frequently diagnosed diseases in older animals [137]. In dogs, while less common than in cats, CKD can similarly result in chronic symptoms and decreased quality of life. A growing body of research suggests that gut dysbiosis and the accumulation of uremic toxins play significant roles in the progression of CKD in animals and humans [42,138]. Dysbiosis can also exacerbate systemic inflammation and oxidative stress, further damaging renal tissues [139]. FMT could serve as a complementary treatment for CKD in animals by targeting gut dysbiosis and improving GI health. By restoring a healthy microbiome, FMT has the potential to reduce the production and absorption of uraemic toxins, alleviate systemic inflammation, and enhance overall gut–kidney axis function. For example, cats with CKD often exhibit elevated levels of uraemic toxins like indoxyl sulfate and p-cresyl sulfate, which originate from gut microbial metabolism [43]. Modulating the microbiome through FMT could help mitigate the effects of these toxins, improving clinical outcomes and slowing disease progression [140]. In dogs, where CKD often coexists with comorbidities such as hypertension and proteinuria, FMT could support renal function by reducing intestinal permeability and systemic endotoxemia [141]. Additionally, as appetite and gastrointestinal disturbances are common in animals with CKD, FMT’s ability to improve gut health could enhance nutrient absorption and overall well-being. While research into the use of FMT in veterinary medicine remains in its early stages, the promising outcomes observed in people with CKD and related conditions suggest that it could provide a novel therapeutic option for managing CKD in dogs and cats.

## 4. Conclusions

These diverse findings highlight the remarkable versatility and therapeutic potential of FMT beyond the gastrointestinal tract in human medicine. While FMT has shown proven efficacy as a standalone treatment for recurrent *C. difficile* infection in humans, its role in companion animals remains less clear-cut. FMT should be considered as an adjunctive therapy rather than a standalone treatment in veterinary medicine, working alongside evidence-based medical interventions. The use of FMT is most appropriate when there is clear evidence of gut dysbiosis accompanying systemic or extraintestinal disorders, reinforcing its role in modulating the gut microbiome to restore health. Highlighting the importance of personalised microbiome-based interventions underscores the need for standardized screening tools to evaluate microbial imbalances in patients. Future research should prioritise controlled clinical trials to explore the efficacy of FMT in managing extraintestinal conditions in dogs and cats. The establishment of species-specific donor screening protocols, adherence to safety standards, and creation of microbiota banks will be critical in translating FMT into routine veterinary practice. Drawing on experiences in human medicine with FMT offers a valuable foundation, but further refinement is necessary to adapt this promising therapeutic approach for domestic animals.

Given the substantial gaps in veterinary knowledge, this review aims to bridge human and veterinary research, using insights from human medicine to inform potential applications in companion animals. However, until controlled trials are conducted in veterinary species, FMT for extraintestinal diseases should be considered an experimental approach rather than an established treatment. Future research should focus on understanding the mechanisms by which FMT exerts systemic effects, developing animal-specific microbiota banks, and determining safe and effective protocols for veterinary applications.

## 5. Future Directions

Despite growing interest in FMT within veterinary medicine, several challenges must be addressed to support its broader implementation. Following the recent publication of the first set of clinical guidelines, FMT remains in its infancy within veterinary medicine. The development of species-specific microbiota banks represents a critical step toward standardisation and wider clinical uptake. Owner awareness and acceptance also present a critical hurdle, as the perception of FMT may influence treatment uptake. Addressing these challenges through targeted research, policy development, and public engagement will be essential to advance the safe and effective application of FMT in veterinary contexts.

Progress is further constrained by the limited volume of published research on gut microbiome alterations in companion animals across a range of disease states. While early studies have begun to highlight microbial shifts in conditions such as atopic dermatitis, metabolic disease, immune-mediated disorders, and hepatic disease, the existing evidence base remains sparse. Investigations into the gut–brain axis in companion animals remain largely unexplored, particularly in relation to behavioural and neurological conditions such as anxiety. Similarly, the role of the gut microbiome in cancer and in extraintestinal infections involving MDROs has received minimal research attention to date. Expanding our understanding of these associations in companion animals is critical to identifying microbial signatures of disease and evaluating the potential of microbiome-targeted therapies, including FMT.

## Figures and Tables

**Table 1:** Published cases of faecal microbiota transplantation (FMT) for the treatment of extra-intestinal disease in people.

Study	Study Design	Disease/Disorder	Participants	Protocol	Clinical Effects	Proposed Translation to Companion Animals	Reference
1	Case report	Alopecia	2 people	N/A	Incidental sustained hair regrowth following FMT for *Clostridioides difficile* infection	Canine and feline alopecia	[22]
2	Case report	Atopic dermatitis	1 person	Endoscopic delivery of FMT into duodenum. Three FMTs administered every other day. Stool from one donor.	Incidental resolution of rash associated with atopic dermatitis, with sustained recovery on follow-up examination	Canine atopic dermatitis	[23]
3	Case report	Atopic dermatitis	9 people	Two doses of 15 frozen, oral capsulized FMTs. Administered over 2 consecutive days. Stool from one donor.	Significant reduction in SCORAD scores following FMT, no significant change with placebo treatment.	Canine atopic dermatitis	[24]
4	Single-centre cohort study	Urinary tract infection caused by MDROs	5 people	At least one FMT, administered either by colonoscopy or oral capsules. Stool from one donor.	FMT application was associated with both the reduced number of MDRO-related urinary tract infections and hospital admission days.	Cainne and feline UTI	[25]
5	Case report	Sepsis and Diarrhoea	1 person	Single fresh FMT delivered via nasogastric tube. Stool from one donor.	Successful treatment of a patient with sepsis and severe diarrhoea after a vagotomy.	Sepsis	[26]
6	Case report	MODS following sepsis	2 people	Single fresh FMT delivered via nasogastric tube. Stool from one donor.	MODS and severe diarrhoea were alleviated in both patients.	Sepsis	[27]
7	Randomized clinical trial	Hepatic encephalopathy	20 people	Single frozen-thawed FMT instilled by rectal enema, retained for 30 min. Stool from one donor.	Significant improvement in PHES and EncephalApp Stroop in the FMT group compared to baseline. No improvement in pre-and post-FMT values among the standard of care arm.	Canine portosystemic shunt, chronic hepatobiliary disease, drug/toxin induced liver injury, feline hepatic lipidosis.	[28]
8	Case series	Recurrent hepatic encephalopathy	10 people	Single FMT instilled via colonoscopy. Stool from one donor.	Sustained clinical response in 6 patients as defined by a significant reduction in arterial ammonia level, and improvement in CTP and MELD scores.	Canine portosystemic shunt, chronic hepatobiliary disease, drug/toxin induced liver injury, feline hepatic lipidosis.	[29]
9	Case report	Epilepsy	1 person	Multiple fresh FMTs administered by gastroscope. Stool from microbiota bank.	Incidental cure of epilepsy in a patient who received FMT for Crohn’s disease, with maintained response 20-months after withdrawing antiepileptic drugs.	Canine epilepsy.	[30]
10	Prospective clinical trial	Unstable diabetes mellitus	14 people	Three washed microbiota transplantations, from frozen-thawed faeces, over 3 days. Instilled into proximal jejunum via gastroendoscopy. Multiple stool donors.	Daily insulin dose and glucose excursions markedly dropped. Glycaemic variability indices significantly improved up to 1 month.	Canine and feline diabetes mellitus.	[31]
11	Randomised controlled trial	T1DM	10 people	Three autologous or allogenic faecal transplantations by nasoduodenal tube using freshly produced faeces at 0, 2, and 4 months.	FMT halted the decline in endogenous insulin production in those recently diagnosed with T1DM.	Canine and feline diabetes mellitus.	[32]
12	Clinical trial	Melanoma	15 people	Single FMT administered via colonoscopy. Stool from one donor.	FMT overcame primary resistance to anti-PD-1 therapy in a subset of patients with advanced melanoma.	Canine and feline refractory melanoma.	[33]
13	Single-arm pilot clinical trial	Systemic lupus erythematosus	20 people	Multiple frozen encapsulated FMTs administered orally for three consecutive weeks (30 capsules per week).	Following FMT there were significant reductions in SLEDAI-2K scores and levels of serum anti-dsDNA antibody.	Canine systemic lupus erythematosus.	[34]
14	Single-centre, double-blind, randomised, placebo-controlled clinical trial	Chronic Kidney Disease	28 people	Multiple frozen encapsulated FMTs administered orally for 30 days. 15 capsules every 12 h four times on day 0, 10, and 30 (total 180 capsules).	More patients in the placebo group progressed to chronic kidney disease than the FMT group. The FMT group maintained stable renal function parameters.	Canine and feline chronic kidney disease.	[35]

Abbreviations: CADESI, Canine Atopic Dermatitis Extent and Severity Index; CTP, Child–Turcotte–Pugh; FMT, faecal microbiota transplantation; MDROs, multidrug-resistant organisms; MELD, model for end-stage liver disease; MODS, multiple organ dysfunction syndrome; PHES, psychometric encephalopathy score; SCORAD, SCORing Atopic Dermatitis; SLEDAI-2K, Systemic Lupus Erythematosus Disease Activity Index 2000; T1DM, type-1 diabetes mellitus.

**Table 2:** Published cases of faecal microbiota transplantation (FMT) for the treatment of extra-intestinal disease in companion animals and rodent models.

Author	Study Design	Disease/Disorder	Participants	Frequency of FMT	Protocol	Clinical Effects	Reference
1	Pilot study, single-arm, open-label clinical trial	CAD	12 dogs	Single	Single fresh FMT delivered orally.	Significant decrease in CADESI scores and PVAS scores following FMT.	[36]
2	Experimental animal study	Depressive behaviour following chronic unpredictable stress	Mice	Single	Fresh FMTs pooled from wild type and NLRP3 KO donor mice, respectively. Antibiotic-treated mice were orally administered FMTs by oral gavage on 3 consecutive days.	Transplantation of gut microbiota from NLRP3 knock-out mice ameliorated chronic unpredictable stress induced depressive-like behaviours.	[37]
3	Experimental animal study	Acute SCI	Mice	Multiple	Fresh FMT from healthy uninjured rats administered by oral gavage once a day on the day of injury and for 2 days after.	FMT prevented SCI induced dysbiosis and anxiety-like behaviour.	[38]
4	Experimental animal study	Obesity	Mice	Single	Frozen FMT from twin mice discordant for obesity. Each FMT was introduced, via a single oral gavage, into a group of 8–9-week-old adult male germ-free C57BL/6J mice.	Increase in total body fat and mass in mice administered FMT from obese donors.	[39]
5	Experimental animal study	T1DM	Mice	Multiple	Multiple fresh FMTs administered via oral gavage into recipient mice four times over 12 days.	FMT from non-obese diabetic mice induced insulitis in non-obese resistant mice.	[40]

Abbreviations: FMT, faecal microbiota transplantation; CAD, canine atopic dermatitis; CADESI, Canine Atopic Dermatitis Extent and Severity Index; PVAS, Pruritus Visual Analog Scale; SCI, spinal cord injury; T1DM, type-1 diabetes mellitus.

## Data Availability

No new data were created or analysed in this study. Data sharing is not applicable to this article.
